# Postoperative Harris Hip Score Versus Harris Hip Score Difference in Hip Replacement: What to Report?

**DOI:** 10.1111/os.14272

**Published:** 2024-10-21

**Authors:** Nikolai Ramadanov, Maximilian Voss, Robert Hable, Hassan Tarek Hakam, Robert Prill, Mikhail Salzmann, Dobromir Dimitrov, Roland Becker

**Affiliations:** ^1^ Center of Orthopaedics and Traumatology University Hospital Brandenburg an der Havel, Brandenburg Medical School Theodor Fontane Brandenburg an der Havel Germany; ^2^ Faculty of Health Science Brandenburg Brandenburg Medical School Theodor Fontane Potsdam Brandenburg Germany; ^3^ Faculty of Applied Computer Science Deggendorf Institute of Technology Deggendorf Germany; ^4^ Department of Surgical Propedeutics, Faculty of Medicine Medical University of Pleven Pleven Bulgaria

**Keywords:** Harris Hip Score, meta‐analysis, patient reported outcome measure, systematic review, total hip arthroplasty

## Abstract

**Background:**

Reliable scientific information is crucial for assessing hip function and evaluating the success of hip surgery. The Harris Hip Score (HHS) is the most widely used tool for measuring hip function and, in particular, the outcomes of hip surgery. The aim of this study was to conduct a systematic review of the literature to identify randomized controlled trials (RCTs) that reported the HHS for hip replacement treatment groups and to test whether there was a substantial difference between reporting only the postoperative HHS or the HHS difference (HHSdiff).

**Methods:**

PubMed, CNKI, and Epistemonikos were searched until March 1, 2024. The risk of bias, level of evidence, and publication bias were assessed. As HHS is a continuous outcome, mean difference (MD) with 95% confidence intervals (CIs) was calculated using the Hartung–Knapp–Sidik–Jonkman method and a common‐effect/random‐effects model. The same approach was used for both postoperative HHS and HHSdiff. The effect of the two treatment groups studied (minimally invasive vs. conventional approach) on postoperative HHS was then compared with the effect of the two groups studied on the difference in HHS.

**Results:**

A total of 41 RCTs, involving 3572 patients, with a low to high risk of bias and a low to moderate publication bias were included. The measured outcome parameters showed a low to moderate level of evidence. There was no relevant difference in the reporting of HHS only postoperatively or HHSdiff when comparing two hip replacement treatment groups in RCTs, measured at 0–0.5, 3, 6, and 12 months postoperatively.

**Conclusion:**

The present study showed that there is no relevant difference between reporting of the HHS only postoperatively or HHSdiff when comparing two hip replacement treatment groups in RCTs. Both methods of HHS reporting produced comparable results in an identical cohort of 3765 patients undergoing hip replacement surgery.

## Graphical abstract

In a study of 41 RCTs, involving 3572 patients, we tried to test whether there was a substantial difference in reporting only the postoperative Harris Hip Score (HHS) or the HHS difference (HHSdiff). We found no relevant difference in reporting of HHS only postoperatively or HHSdiff when comparing two hip replacement treatment groups in RCTs, measured at 0–0.5, 3, 6, and 12 months postoperatively. The present study showed that there is no relevant difference in the reporting of the HHS postoperatively only or the HHSdiff when comparing two hip replacement treatment groups in RCTs. Both methods of HHS reporting produced comparable results in an identical cohort of 3765 patients undergoing hip replacement surgery.

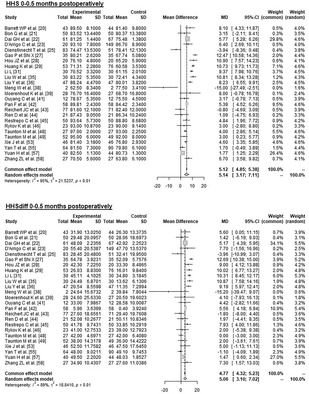

**Abbreviations**
CI: confidence interval
HHA: hip hemiarthroplasty
HHS: Harris Hip Score
ITT: intention to treat
MCID: minimum clinically important difference
MD: mean difference
PRISMA: Preferred Reporting Items for Systematic Reviews and Meta‐Analysis
PROM: patient‐reported outcome measure
PROSPERO: International Prospective Register of Systematic Reviews
RCT: randomized controlled trial
RoB: risk of bias
SF‐36: Short Form‐36
THA: total hip arthroplasty
WOMAC: Western Ontario McMaster Osteoarthritis Index

## 1 Introduction

Millions of patients with various hip pathologies have been relieved of pain after hip arthroplasty, with varying functional outcomes. To evaluate the success of hip surgery, it is essential that science provides us with reliable information to assess hip function. In addition to the use of performance or time‐based measures, patient‐reported outcome measures (PROMs) have been mainly used to capture this domain [1]. The Harris Hip Score (HHS) [2] is the most commonly used tool to measure hip function and, in particular, hip surgery outcomes. Several studies have tested the validity and reliability of the HHS and found it to be a valid instrument that provides reliable information about the functional outcome of total hip arthroplasty (THA) [3, 4, 5, 6, 7, 8, 9, 10]. In fact, the HHS is becoming established as the gold standard [11]. It is often used as a reference for assessing the validity of other PROMs for hip function [11]. The HHS outperforms the Western Ontario McMaster Osteoarthritis Index (WOMAC) [10], the Short Form‐36 (SF‐36) [10, 12, 13], and the simple assessment of walking speed [13]. When using the HHS, the overall score is derived from the assessment of four aspects: pain, function, degree of deformity, and hip range of motion. The better the functional hip outcome, the higher the total score, which ranges from 0 to 100 points. Authors of orthopedic literature regularly use the calculated results of the HHS in randomized controlled trials (RCTs) to decide which treatment is superior for hip surgery. After all, RCTs of THA, in which hip function is so often described using the HHS, form the top of the evidence‐based medicine pyramid.

However, in the orthopedic literature, most RCTs of THA only report the effect of postoperative HHS between two THA treatment groups. Some higher‐quality RCTs also report preoperative HHS, and a few RCTs even compare the two THA treatment groups by looking at the HHS difference (HHSdiff). The HHSdiff is defined as the difference between preoperative and postoperative HHS values. Reporting the HHSdiff is likely to provide more accurate results and sufficient insight into the true effect in terms of relative treatment effect. Otherwise, there is no information on whether both treatment groups started with comparable preoperative HHS or whether one group had better preoperative hip function than the other. The counter‐argument is that the preoperative condition of the hip cannot be that relevant, as the entire hip joint is being replaced. However, we still do not know what is better to report: is it sufficient to report only the postoperative HHS or is it necessary to report the HHSdiff? Do the two different reports lead to relevant differences that might lead to different conclusions when comparing hip replacement treatment groups?

The aim of this study was to perform a systematic review of the literature to find RCTs that reported HHS of hip replacement treatment groups and to test whether there was a substantial difference in reporting only the postoperative HHS or the HHSdiff.

## 2 Methods

This study protocol was registered in the International Prospective Register of Systematic Reviews (PROSPERO) on February 27, 2024 [CRD42024513554]. We strictly followed author guidelines for conducting systematic reviews and meta‐analyses during the production [14], and the updated version of the Preferred Reporting Items for Systematic Reviews and Meta‐Analysis (PRISMA) guidelines [15] in reporting this review. The PRISMA checklist is available in the Supplementary Appendix.

### 2.1 Systematic Review

PubMed, CNKI, and Epistemonikos were searched until March 1, 2024. A Boolean search strategy was constructed and adapted to the syntax of the databases used. The search term used was (((total hip arthroplasty) OR (hemiarthroplasty) OR (THA) OR (HA) OR (hip replacement)) AND ((conventional approach) OR (DAA) OR (anterior approach) OR (AMIS) OR (ALMI) OR (SuperPATH) OR (minimally invasive))). No publication language restrictions were applied. A stepwise screening process was performed according to the PRISMA guidelines [15]. First, the titles and abstracts of the identified records were screened. The full texts of the screened articles were then assessed for eligibility. The decision to include each study was made by consensus between two reviewers (NR and MV). The agreement between the reviewers was measured using the kappa coefficient (*κ*). The following inclusion criteria were applied: (i) only RCTs were included, (ii) human participants with hip disease or fracture, (iii) who underwent hip replacement by THA or hip hemiarthroplasty (HHA) using a minimally invasive hip approach/technique compared to a conventional approach/technique, and (iv) both the minimally invasive and conventional treatment groups must report pre‐ and postoperative functional hip outcome as measured by the HHS. The following exclusion criteria were applied: (i) robotic assistance and computer navigation, (ii) revision surgery, (iii) dual mobility THA, (iv) monopolar HHA, and (v) missing outcome data.

### 2.2 Data Extraction

Data extraction was performed independently by two reviewers (NR and MV), and disagreements were resolved by consensus, collecting data on RCT characteristics, methods, quality assessment, participant characteristics, details of interventions, relevant outcomes, and relevant additional information. The extracted data are available in the Supplementary Appendix. Missing standard deviations were imputed. We adhered to the intention‐to‐treat (ITT) analysis for data extraction from RCTs. HHS was measured preoperatively and at different time points postoperatively: 0–1.5, 3, 6, and ≥ 12 months.

### 2.3 Quality Assessment

Risk of bias (RoB) and level of evidence were assessed by two reviewers independently using the Cochrane RoB 2 tool [16] and the GRADE system recommendations [17], with disagreements resolved by consensus. Publication bias was calculated using Begg's and Egger's tests.

### 2.4 Statistics

As HHS is a continuous outcome, the mean difference (MD) with 95% confidence intervals (CIs) was calculated using the Hartung–Knapp–Sidik–Jonkman method and a common‐effect/random‐effects model. The same approach was used for both postoperative HHS and HHSdiff. Before this, the HHSdiff was calculated by subtracting the preoperative HHS value from the postoperative HHS value for each RCT. The heterogeneity of the RCTs was assessed using the Cochrane *Q* test (*p* value < 10 indicates heterogeneity) and the Higgins test *I* ^2^ (low heterogeneity < 25%, moderate heterogeneity: 25%–75%, and high heterogeneity > 75%) [18]. As these values indicate a high degree of heterogeneity for some parameters, we have retained the random‐effects model in our presentation of the results. Studies were weighted using inverse variance. Minimally invasive hip replacement was referred to as the experimental group, and conventional hip replacement was referred to as the control group. The effect of the two treatment groups studied (minimally invasive vs. conventional approach) on postoperative HHS was then compared with the effect of the two groups studied on the difference in HHS. A professional statistician (RH) performed all statistical calculations using the R packages meta and metafor.

## 3 Results

### 3.1 Systematic Review

**FIGURE 1:**
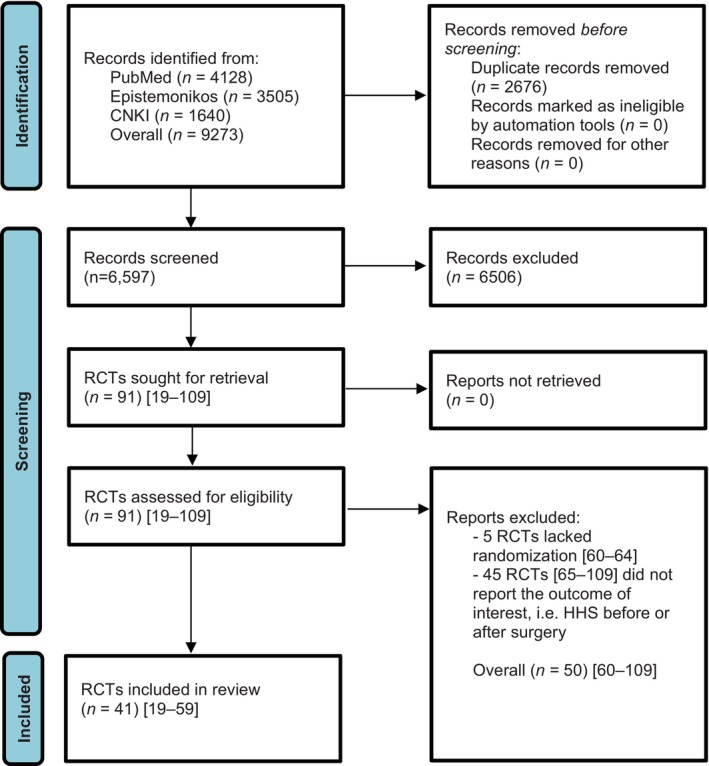
PRISMA flowchart.

The study selection process is illustrated in a PRISMA flowchart (Figure 1). After excluding duplicates, the initial literature search yielded 6597 records. A total of 91 RCTs [19, 20, 21, 22, 23, 24, 25, 26, 27, 28, 29, 30, 31, 32, 33, 34, 35, 36, 37, 38, 39, 40, 41, 42, 43, 44, 45, 46, 47, 48, 49, 50, 51, 52, 53, 54, 55, 56, 57, 58, 59, 60, 61, 62, 63, 64, 65, 66, 67, 68, 69, 70, 71, 72, 73, 74, 75, 76, 77, 78, 79, 80, 81, 82, 83, 84, 85, 86, 87, 88, 89, 90, 91, 92, 93, 94, 95, 96, 97, 98, 99, 100, 101, 102, 103, 104, 105, 106, 107, 108, 109] were screened by full‐text analysis. Of these 91 RCTs [19, 20, 21, 22, 23, 24, 25, 26, 27, 28, 29, 30, 31, 32, 33, 34, 35, 36, 37, 38, 39, 40, 41, 42, 43, 44, 45, 46, 47, 48, 49, 50, 51, 52, 53, 54, 55, 56, 57, 58, 59, 60, 61, 62, 63, 64, 65, 66, 67, 68, 69, 70, 71, 72, 73, 74, 75, 76, 77, 78, 79, 80, 81, 82, 83, 84, 85, 86, 87, 88, 89, 90, 91, 92, 93, 94, 95, 96, 97, 98, 99, 100, 101, 102, 103, 104, 105, 106, 107, 108, 109], 50 RCTs [60, 61, 62, 63, 64, 65, 66, 67, 68, 69, 70, 71, 72, 73, 74, 75, 76, 77, 78, 79, 80, 81, 82, 83, 84, 85, 86, 87, 88, 89, 90, 91, 92, 93, 94, 95, 96, 97, 98, 99, 100, 101, 102, 103, 104, 105, 106, 107, 108, 109] with full interrater agreement (*κ* = 1.0) were excluded for the following reasons: five RCTs lacked randomization [60, 61, 62, 63, 64] and 45 RCTs [65, 66, 67, 68, 69, 70, 71, 72, 73, 74, 75, 76, 77, 78, 79, 80, 81, 82, 83, 84, 85, 86, 87, 88, 89, 90, 91, 92, 93, 94, 95, 96, 97, 98, 99, 100, 101, 102, 103, 104, 105, 106, 107, 108, 109] did not report the outcome of interest, that is, HHS before or after surgery. A total of 41 RCTs [19, 20, 21, 22, 23, 24, 25, 26, 27, 28, 29, 30, 31, 32, 33, 34, 35, 36, 37, 38, 39, 40, 41, 42, 43, 44, 45, 46, 47, 48, 49, 50, 51, 52, 53, 54, 55, 56, 57, 58, 59] with 3572 patients were included in the final meta‐analysis. Of these patients, 1720 received minimally invasive hip replacement and 1852 received conventional hip replacement. The main characteristics of the patients and the included RCTs are listed in Table 1.

**TABLE 1:** Main characteristics of RCTs.

RCT	Year of publication, origin	Patients, N	THA/HHA	Approach	Osteoarthrosis, N	Femoral neck fracture, N	Dysplasia, N	ANFH, N
Auffarth et al. [19]	2011, Austria	24	HHA	MI DAA	0	24	0	0
		24		CA LA	0	24	0	0
Barrett, Turner, and Leopold [20]	2013, USA	43	THA	MI DAA	43	0	0	0
		44		CA PL	44	0	0	0
Bon et al. [21]	2019, France	50	THA	MI DAA	50	0	0	0
		50		CA P	50	0	0	0
Dai, Yin, Ji, and Yi [22]	2019, China	61	HHA	MI S	0	61	0	0
		67		CA PL	0	67	0	0
D'Arrigo et al. [23]	2009, Italy	20	THA	MI DAA	20	0	0	0
		149		CA L	149	0	0	0
De Anta‐Diaz et al. [24]	2016, Spain	49	THA	MI DAA	49	0	0	0
		50		CA L	50	0	0	0
Dienstknecht et al. [25]	2013, Germany	83	THA	MI MH	83	0	0	0
		51		CA L	51	0	0	0
Ding, Li, and Luo [26]	2023, China	43	HHA	MI S	0	43	0	0
		43		CA L	0	43	0	0
Gao and Shi [27]	2020, China	35	THA	MI S	0	35	0	0
		35		CA P	0	35	0	0
Hou, Bao, and Cheng [28]	2017, China	20	THA	MI S	6	0	0	14
		20		CA	5	0	0	15
Huang et al. [29]	2021, China	53	THA	MI S	0	16	0	37
		76		CA L	0	18	0	58
Jia et al. [30]	2017, China	32	HHA	MI S	0	32	0	0
		32		CA P	0	32	0	0
Li [31]	2020, China	30	THA	MI S	NR	NR	NR	NR
		30		CA PL	NR	NR	NR	NR
Li [32]	2021, China	41	HHA	MI S	0	41	0	0
		41		CA PL	0	41	0	0
Li et al. [33]	2023, China	35	THA	MI S	0	0	0	35
		30		CA PL	0	0	0	30
Ling, Zhou, and Fu [34]	2020, China	50	THA	MI S	0	50	0	0
		50		CA PL	0	50	0	0
Liu et al. [35]	2022, China	30	THA	MI S	3	13	0	14
		30		CA	6	9	0	15
Liu et al. [36]	2021, China	47	THA	MI S	0	47	0	0
		47		CA PL	0	47	0	0
Martin et al. [37]	2011, Belgium	42	THA	MI AL	37	0	0	5
		41		CA L	37	0	0	4
Meng et al. [38]	2020, China	2	THA	MI S	0	0	0	4
		2		CA PL	0	0	0	4
Moerenhout et al. [39]	2019, Canada	28	THA	MI DAA	NR	0	0	NR
		27		CA P	NR	0	0	NR
Müller et al. [40]	2010, Germany	21	THA	MI AL	21	0	0	0
		16		CA L	16	0	0	0
Ouyang et al. [41]	2018, China	12	THA	MI S	5	0	0	7
		12		CA PL	6	0	0	6
Pan et al. [42]	2020, China	58	THA	MI S	12	26	NR	15
		58		CA PL	11	25	NR	18
Reichert et al. [43]	2018, Germany	77	THA	MI DAA	77	0	0	0
		71		CA L	71	0	0	0
Ren et al. [44]	2016, China	21	THA	MI S	0	0	0	21
		21		CA	0	0	0	21
Restrepo, Parvizi, Pour, and Hozack [45]	2010, USA	50	THA	MI DAA	50	0	0	0
		50		CA L	50	0	0	0
Rykov et al. [46]	2017, Netherlands	23	THA	MI DAA	23	0	0	0
		23		CA PL	23	0	0	0
Schwarze et al. [47]	2017, Germany	22	THA	MI AL	22	0	0	0
		21		CA L	21	0	0	0
Taunton, Mason, Odum, and Springer [48]	2014, USA	27	THA	MI DAA	27	0	0	0
		27		CA P	27	0	0	0
Taunton et al. [49]	2018, USA	52	THA	MI DAA	52	0	0	0
		49		CA P	49	0	0	0
Varela‐Egocheaga et al. [50]	2013, Spain	25	THA	MI L	21	0	0	4
		25		CA L	22	0	0	3
Wang and Tian [51]	2021, China	50	HHA	MI S	0	50	0	0
		50		CA PL	0	50	0	0
Wang and Ge [52]	2021, China	43	THA	MI S	0	43	0	0
		42		CA PL	0	42	0	0
Xie et al. [53]	2017, China	46	THA	MI S	46	0	0	0
		46		CA P	46	0	0	0
Xu, Hu, and Yang [54]	2018, China	46	HHA	MI S	0	46	0	0
		46		CA P	0	46	0	0
Yan et al. [55]	2017, China	64	THA	MI S	14	11	0	39
		90		CA L	12	23	0	55
Yang et al. [56]	2010, China	55	THA	MI AL	12	11	0	32
		55		CA PL	19	13	0	23
Yuan, Zhu, Sun, and Zhang [57]	2018, China	40	THA	MI S	5	21	4	10
		44		CA PL	6	24	2	12
Zhang, Lin, and Xia [58]	2019, China	27	THA	MI S	7	0	5	15
		27		CA PL	9	0	4	14
Zhao et al. [59]	2017, China	60	THA	MI DAA	41	0	6	13
		60		CA PL	40	0	7	13

Abbreviations: AL, anterolateral; ANFH, avascular necrosis of the femoral head; CA, conventional approach; DAA, direct anterior approach; HHA, hip hemiarthroplasty; L, lateral; MH, MicroHip; MI, minimally invasive; NR, not reported; P, posterior; PL, posterolateral; S, SuperPATH; THA, total hip arthroplasty.

### 3.2 Quality Assessment

The RoB assessment is shown in Table 2, where 14 RCTs [21, 23, 24, 25, 30, 35, 38, 39, 41, 43, 45, 53, 56, 59] were considered to have a low RoB, 14 RCTs [19, 22, 26, 28, 29, 33, 37, 40, 48, 49, 51, 54, 55, 58] have a moderate RoB, and 13 RCTs [20, 27, 31, 32, 34, 36, 42, 44, 46, 47, 50, 52, 57] have a high RoB. The level of evidence is shown in Table 3, with low‐quality evidence for HHS 0–1.5, 3, and 6 months postoperatively and moderate‐quality evidence for HHS 12 months postoperatively. The assessment of publication bias using Begg's and Egger's tests is shown in Table 4. The corresponding funnel plots are shown in the Supplementary Appendix.

**TABLE 2:** Risk of bias assessment.

RCT	Bias arising from the randomization process	Bias due to deviation from intended interventions	Bias due to missing outcome data	Bias in measurement of the outcome	Bias in selection of the reported result	Overall risk of bias
Auffarth et al. [19]	+	?	?	+	+	?
Barrett, Turner, and Leopold [20]	+	−	?	?	+	−
Bon et al. [21]	+	+	+	+	+	+
Dai, Yin, Ji, and Yi [22]	+	+	?	+	+	?
D'Arrigo et al. [23]	+	+	+	+	+	+
De Anta‐Diaz et al. [24]	+	+	+	+	+	+
Dienstknecht et al. [25]	+	+	+	+	+	+
Ding, Li, and Luo [26]	+	+	?	?	+	?
Gao and Shi [27]	+	?	−	+	+	−
Hou, Bao, and Cheng [28]	+	?	+	+	+	?
Huang et al. [29]	+	?	+	+	+	?
Jia et al. [30]	+	+	+	+	+	+
Li [31]	+	?	−	−	+	−
Li [32]	+	+	−	+	+	−
Li et al. [33]	+	+	?	+	+	?
Ling, Zhou, and Fu [34]	+	+	−	+	+	−
Liu et al. [35]	+	+	+	+	+	+
Liu et al. [36]	+	+	−	+	+	−
Martin et al. [37]	+	?	+	+	?	?
Meng et al. [38]	+	+	+	+	+	+
Moerenhout et al. [39]	+	+	+	+	+	+
Müller et al. [40]	+	+	?	?	+	?
Ouyang et al. [41]	+	+	+	+	+	+
Pan et al. [42]	+	?	−	+	+	−
Reichert et al. [43]	+	+	+	+	+	+
Ren et al. [44]	+	?	−	?	?	−
Restrepo, Parvizi, Pour, and Hozack [45]	+	+	+	+	+	+
Rykov et al. [46]	+	+	−	+	+	−
Schwarze et al. [47]	+	?	−	−	+	−
Taunton, Mason, Odum, and Springer [48]	+	+	?	+	+	?
Taunton et al. [49]	+	+	?	+	+	?
Varela‐Egocheaga et al. [50]	+	−	+	+	+	−
Wang and Tian [51]	+	+	?	?	+	?
Wang and Ge [52]	+	?	−	+	+	−
Xie et al. [53]	+	+	+	+	+	+
Xu, Hu, and Yang [54]	+	+	?	?	+	?
Yan et al. [55]	+	?	?	+	+	?
Yang et al. [56]	+	+	+	+	+	+
Yuan, Zhu, Sun, and Zhang [57]	+	?	−	+	+	−
Zhang, Lin, and Xia [58]	+	+	?	+	+	?
Zhao et al. [59]	+	+	+	+	+	+

Abbreviations: +, low risk of bias; ?, some concerns; −, high risk of bias; RCT, randomized controlled trial.

**TABLE 3:** Level of evidence assessment according to GRADE recommendations.

No. of studies	Design	Risk of bias	Inconsistency	Indirectness	Imprecision	Other considerations	Quality of evidence
HHS postoperatively and HHS difference							
1. HHS 0–1.5 months postoperatively							
	RCT	Moderate	Serious inconsistency	No serious indirectness	No serious imprecision	—	Low
2. HHS 3 months postoperatively							
	RCT	Moderate	Serious inconsistency	No serious indirectness	No serious imprecision	—	Low
3. HHS 6 months postoperatively							
	RCT	Moderate	Serious inconcistency	No serious indirectness	No serious imprecision	—	Low
4. HHS 12 months postoperatively							
	RCT	Moderate	No serious inconcistency	No serious indirectness	No serious imprecision	—	Moderate

Abbreviations: HHS, Harris Hip Score; RCT, randomized controlled trial.

**TABLE 4:** Overview of the most important results of the meta‐analysis.

HHS	RCTs, N	Patients, N	Treatment effect	*p*	*I* ^2^	Tau^2^	Egger bias	Egger *p*
HHS 0–1, 5 months postoperatively	25	2145	5.14	< 0.0001***	0.95	21.52	0.81	0.5556
HHSdiff 0–1, 5 months postoperatively	25	2145	5.06	< 0.0001***	0.89	18.84	0.43	0.6285
HHS 3 months postoperatively	25	2094	3.34	0.0010***	0.93	16.77	1.48	0.1792
HHSdiff 3 months postoperatively	25	2094	3.59	0.0009***	0.85	17.80	0.63	0.4064
HHS 6 months postoperatively	19	1494	2.56	0.0071**	0.86	10.40	1.60	0.0878
HHSdiff 6 months postoperatively	19	1494	3.07	0.0051**	0.79	12.10	1.27	0.0688
HHS ≥ 12 months postoperatively	17	1242	1.11	0.0068**	0.08	1.70	0.64	0.1888
HHSdiff ≥ 12 months postoperatively	17	1242	0.60	0.3918	0.07	4.73	0.36	0.4228

Abbreviations: HHS, Harris Hip Score; HHSdiff, Harris Hip Score difference; RCT, randomized controlled trials.

*significant; **highly significant; ***very highly significant.

### 3.3 Meta‐Analysis of HHS Postoperatively and HHS Difference

#### 3.3.1 At 0–1.5 Months Postoperatively

**FIGURE 2:**
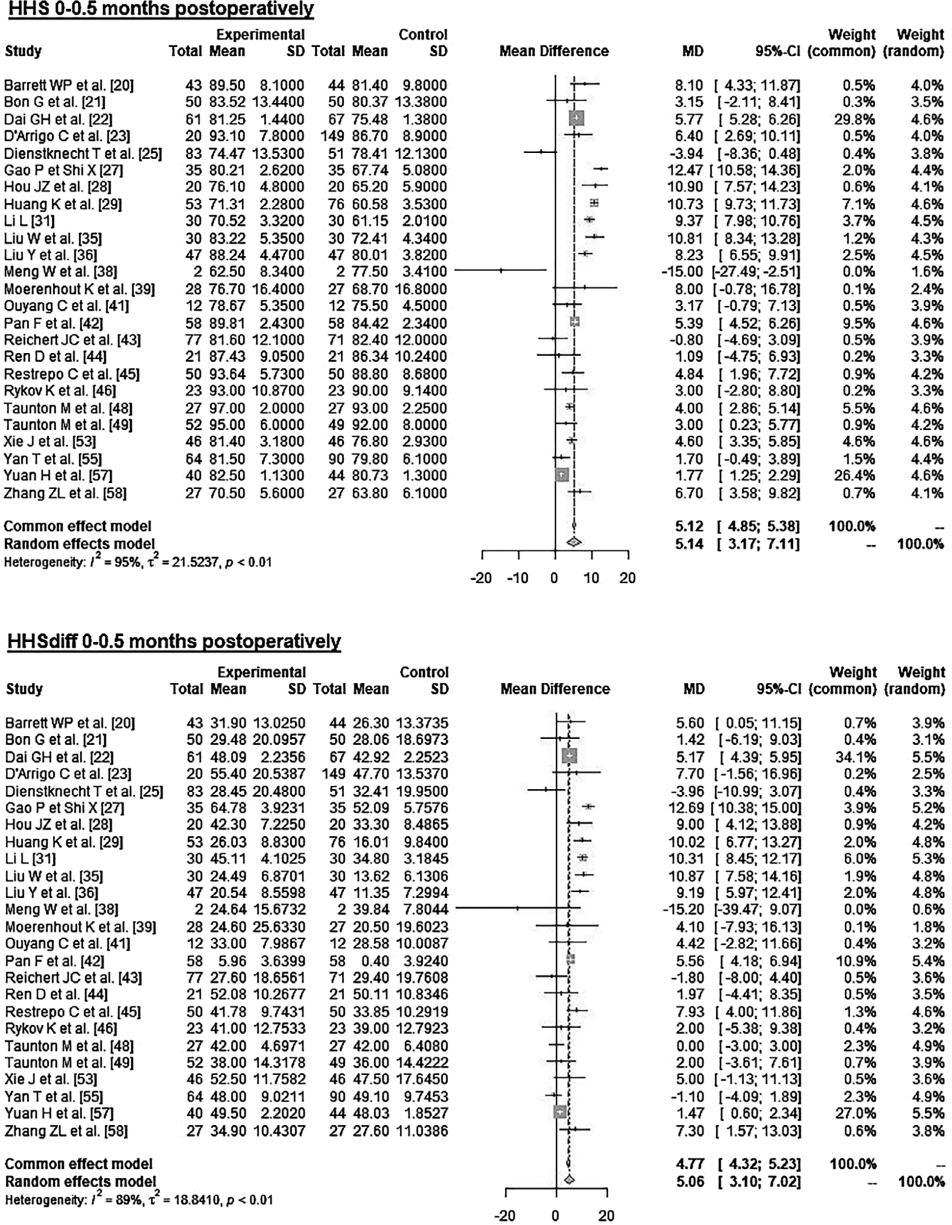
Forest plot of the HHS 0–1.5 months postoperatively. CI, confidence interval; HHS, Harris Hip Score; MD, mean difference; SD, standard deviation.

Data on 2145 patients from 25 RCTs were pooled (Figure 2 and Table 4), comparing both treatment groups by HHS 0–1.5 months postoperatively and HHSdiff 0–1.5 months postoperatively. The experimental group showed 5.14 points higher HHS 0–1.5 months postoperatively (MD = 5.14, 95% CI 3.17–7.11). The experimental group showed 5.06 points higher HHSdiff 0–1.5 months postoperatively (MD = 5.06, 95% CI 3.10–7.02).

#### 3.3.2 At 3 Months Postoperatively

**FIGURE 3:**
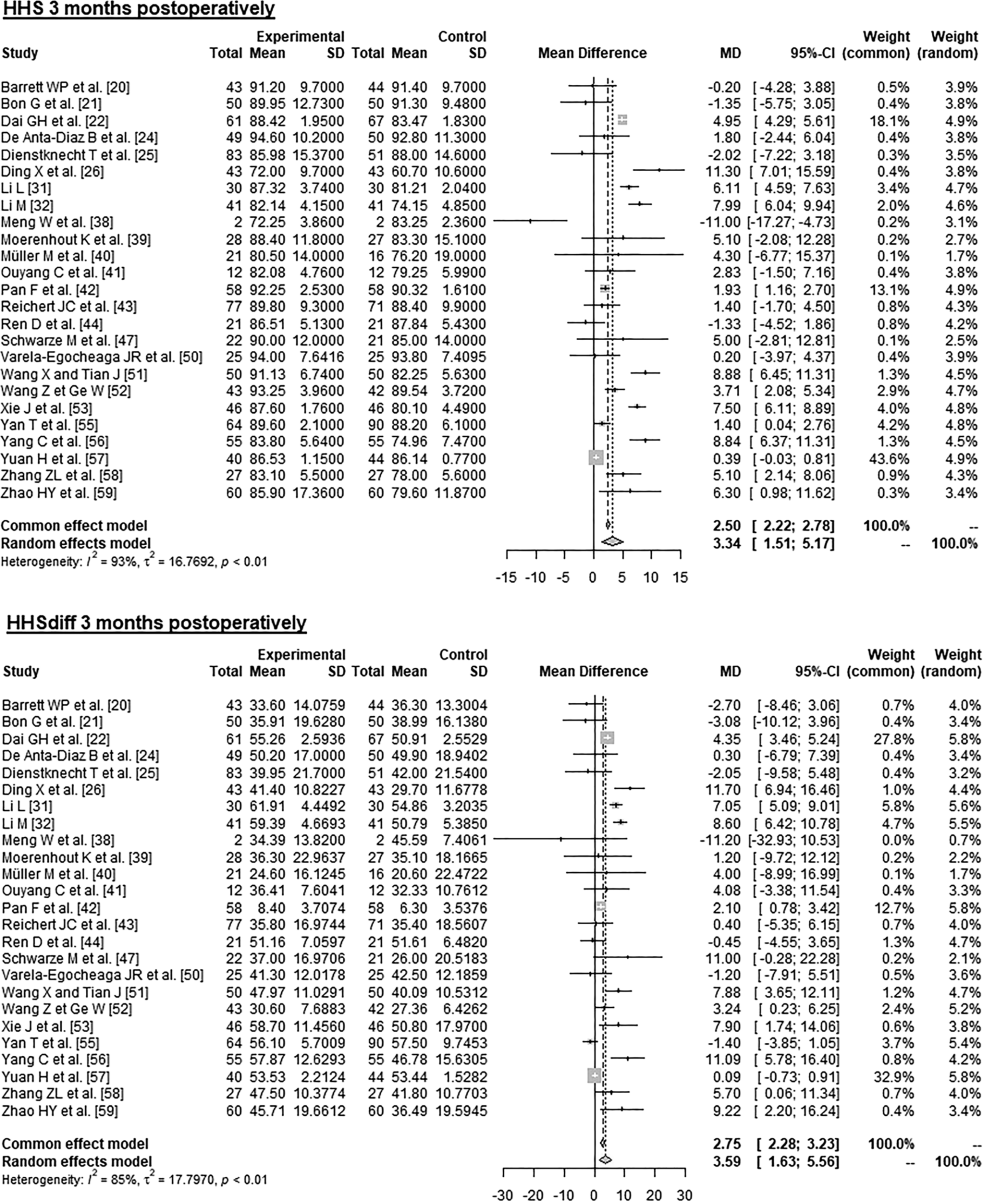
Forest plot of the HHS 3 months postoperatively. CI, confidence interval; HHS, Harris Hip Score; MD, mean difference; SD, standard deviation.

Data on 2094 patients from 25 RCTs were pooled (Figure 3 and Table 4), comparing both treatment groups by HHS 3 months postoperatively and HHSdiff 3 months postoperatively. The experimental group showed 3.34 points higher HHS 3 months postoperatively (MD = 3.34, 95% CI 1.51–5.17). The experimental group showed 3.59 points higher HHSdiff 3 months postoperatively (MD = 3.59, 95% CI 1.63–5.56).

#### 3.3.3 At 6 Months Postoperatively

**FIGURE 4:**
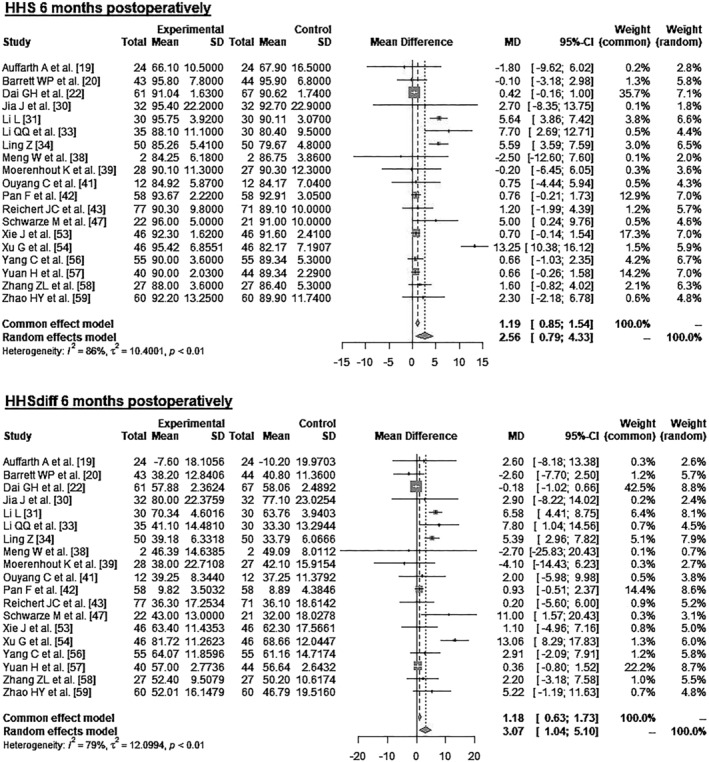
Forest plot of the HHS 6 months postoperatively. CI, confidence interval; HHS, Harris Hip Score; MD, mean difference; SD, standard deviation.

Data on 1528 patients from 19 RCTs were pooled (Figure 4 and Table 4), comparing both treatment groups by HHS 6 months postoperatively and HHSdiff 6 months postoperatively. The experimental group showed 2.56 points higher HHS 6 months postoperatively (MD = 2.56, 95% CI 0.79–4.33). The experimental group showed 3.07 points higher HHSdiff 6 months postoperatively (MD = 3.07, 95% CI 1.04–5.10).

#### 3.3.4 At 12 Months Postoperatively

**FIGURE 5:**
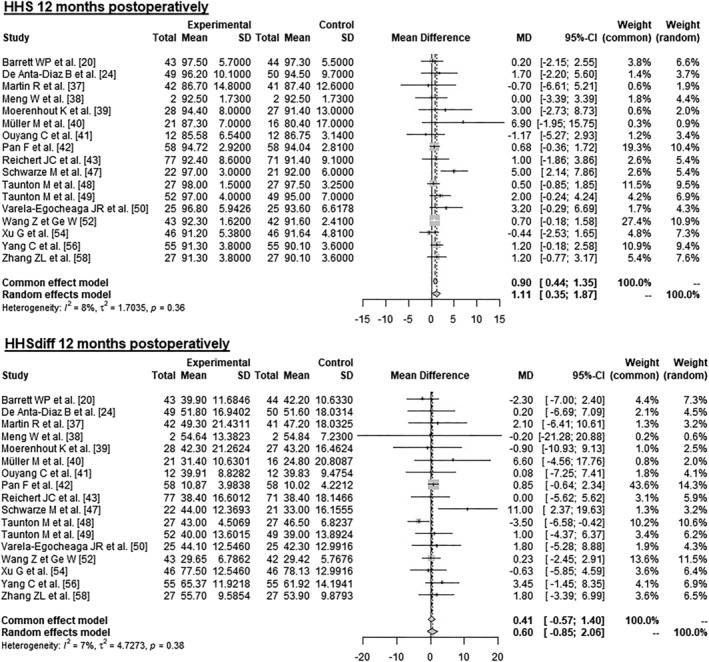
Forest plot of the HHS 12 months postoperatively. CI, confidence interval; HHS, Harris Hip Score; MD, mean difference; SD, standard deviation.

Data on 1188 patients from 17 RCTs were pooled (Figure 5 and Table 4), comparing both treatment groups by HHS 12 months postoperatively and HHSdiff 12 months postoperatively. The experimental group showed 1.11 points higher HHS 12 months postoperatively (MD = 1.11, 95% CI 0.35–1.87). There was no significant difference in HHSdiff 12 months postoperatively (MD = 0.60, 95% CI −0.85–2.06).

## 4 Discussion

This systematic review and meta‐analysis investigated whether the difference in HHS resulted in a different postoperative treatment effect than postoperative HHS alone. The study was based on a comparison of the treatment effect of 41 RCTs with a total of 3572 patients in which minimally invasive THA/HHA was compared with conventional THA/HHA. The HHSdiff was defined as the difference between the preoperative HHS and the HHS measured after surgery. The main finding of this study was that there was no relevant difference between reporting of HHS only postoperatively or the HHSdiff when comparing two hip replacement treatment groups in RCTs.

The HHS is a proven evaluation system for assessing the clinical condition of the hip, particularly after hip replacement surgery [2]. The HHS was developed in the 1960s by William H. Harris [2]. The HHS is divided into four main categories: pain (44 points), function (47 points, divided into daily activities and walking ability), range of motion (5 points), and deformity (4 points) [2]. The maximum score is 100, with higher scores indicating a better condition of the hip. Scores above 90 are considered excellent, 80–89 as good, 70–79 as fair, and below 70 as poor [2]. In clinical use, the HHS is a valuable tool for evaluating outcomes after hip surgery, particularly for hip arthroplasty [2]. Orthopedic surgeons use the HHS to track patient progress, evaluate the success of operations, and support treatment decisions. The standardized assessment allows us to collect objective data on the hip condition before and after surgery, which is important for both clinical research and individual patient care. In addition, the HHS allows comparison of results between different clinics and surgical methods, which helps to improve surgical techniques and patient care. The HHS is particularly useful as it integrates both subjective patient assessments and objective clinical findings, providing a comprehensive assessment of hip health.

The main finding of this meta‐analysis has important implications for the scientific literature on hip arthroplasty and hip function in general as well as for orthopedic practice. The numerous RCTs and meta‐analyses that reported only postoperative HHS are reliable, because even when the preoperative HHS, i.e. HHSdiff, is taken into account, no other relevant conclusions can be drawn. In other words, even if the orthopedic literature ignores preoperative HHS, it still seems to provide valid results when comparing two hip replacement treatment groups. It is also important for the practical work of the orthopedic surgeon to know that preoperative HHS does not seem to have a decisive influence on postoperative HHS. In other words, the postoperative outcome is more likely to be dependent on surgery‐related parameters such as correct implant positioning, soft tissue trauma, or surgeon experience.

To be precise, there was a slight difference between postoperative HHS and HHSdiff 12 months postoperatively. The experimental group showed a 1.11 point higher HHS 12 months postoperatively, but there was no significant difference in HHSdiff 12 months postoperatively. First, it should be emphasized that a difference of 1.11 is not clinically relevant, as the smallest minimum clinically important difference (MCID) for HHS reported in the literature is 7.9 [110]. Furthermore, the difference between postoperative HHS and HHSdiff 12 months postoperatively may simply be due to the sample size of the included primary studies and patients. This finding should be verified with a larger sample size.

Due to the uniqueness of the present study, a direct comparison with the literature is not possible. Previous studies have shown that the HHS is a valid tool that provides reliable information on the functional outcome of THA [3, 4, 5, 6, 7, 8, 9, 10] and is increasingly establishing itself as the gold standard [11]. Together with the results of this meta‐analysis, the use of the HHS should be further expanded.

Strengths and limitations should be mentioned: (i) This meta‐analysis was conducted and reported according to (PRISMA) guidelines (ii) and high‐quality statistical methods were used. (iii) The meta‐analysis was restricted to include only RCTs known to have a very good methodological design. (iv) The inclusion of a total of 41 RCTs with 3572 patients is a very large sample, which allows more reliable and generalizable conclusions to be drawn. (v) Of the 41 RCTs included in this meta‐analysis, 25 (61%) were from China. It has to be regarded as an advantage of this systematic review that it did not ignore the huge database of the CNKI. (vi) The main limitation of this meta‐analysis is that the treatment groups were limited to THA or HHA. It is not known whether the same study performed in patients undergoing, for example, hip arthroscopy or hip osteotomy might give different results. (vii) The direct comparison of the treatment effects of postoperative HHS and HHSdiff was a simple head‐to‐head comparison. There is no statistical test to make an objective calculation. (viii) In similar studies with such a large amount of data extraction, there is always a risk of extracting incorrect figures.

## 5 Conclusion

This study shows that there is no relevant difference between reporting the HHS postoperatively only or the HHSdiff when comparing two hip replacement treatment groups in RCTs. Both methods of HHS reporting produced comparable results in an identical cohort of 3765 patients undergoing hip replacement surgery.

## Author Contributions

N.R. and M.V. performed the systematic literature review and data extraction. N.R. and H.T.H. performed the quality assessment of the included studies. R.H. carried out all statistical calculations. N.R. prepared all tables and figures. All authors supervised the work and read the final version.

## Ethics Statement

The authors have nothing to report.

## Consent

The authors have nothing to report.

## Conflicts of Interest

The authors declare no conflicts of interest.

## Supporting information


**Data S1.** Supporting Information.

## Data Availability

The raw data extraction sheet is available in the supplement.

## References

[1] J. Kirschner , S. Michel , R. Becker , et al., “Determination of Relationships Between Symmetry‐Based, Performance‐Based, and Functional Outcome Measures in Patients Undergoing Total Hip Arthroplasty,” Journal Personalised Medicine 13, no. 7 (2023): 1046, 10.3390/jpm13071046. PMC10381123 37511659

[2] W. H. Harris , “Traumatic Arthritis of the Hip After Dislocation and Acetabular Fractures: Treatment by Mold Arthroplasty. An End‐Result Study Using a New Method of Result Evaluation,” Journal of Bone and Joint Surgery. American Volume 51, no. 4 (1969): 737–755. 5783851

[3] P. Söderman and H. Malchau , “Is the Harris Hip Score System Useful to Study the Outcome of Total Hip Replacement?,” Clinical Orthopaedics and Related Research 384 (2001): 189–197. 10.1097/00003086-200103000-00022 11249165

[4] M. J. Bryant , J. R. Kernohan , J. R. Nixon , and R. A. B. Mollan , “A Statistical Analysis of Hip Scores,” Journal of Bone and Joint Surgery 75 (1993): 705–709. 10.1302/0301-620X.75B5.8376424 8376424

[5] A. Laupacis , R. Bourne , C. Rorabeck , et al., “The Effect of Elective Total Hip Replacement on Health‐Related Quality of Life,” Journal of Bone and Joint Surgery 75 (1993): 1619–1626. 10.2106/00004623-199311000-00006 8245054

[6] M. Sullivan , J. Karlsson , and J. R. Ware , “The Swedish SF‐36 Health Survey—I. Evaluation of Data Quality, Scaling Assumptions, Reliability and Construct Validity Across General Populations in Sweden,” Social Science & Medicine 41 (1995): 1349–1358. 8560302 10.1016/0277-9536(95)00125-q

[7] P. Soderman and H. Malchau , “Is the Harris Hip Score System Useful to Study the Outcome of Total Hip Replacement?,” Clinical Orthopaedics and Related Research 384 (2001): 189–197. 10.1097/00003086-200103000-00022 11249165

[8] P. Soderman , H. Malchau , and P. Herberts , “Outcome of Total Hip Replacement: A Comparison of Different Measurement Methods,” Clinical Orthopaedics and Related Research 390 (2001): 163–172. 10.1097/00003086-200109000-00019 11550862

[9] B. F. Kavanagh and R. H. Fitzgerald, Jr. , “Clinical and Roentgenographic Assessment of Total Hip Arthroplasty. A New Hip Score,” Clinical Orthopaedics and Related Research 193 (1985): 133–140. 3971612

[10] J. G. Wright and N. L. Young , “A Comparison of Different Indices of Responsiveness,” Journal of Clinical Epidemiology 50, no. 3 (1997): 239–246. 9120522 10.1016/s0895-4356(96)00373-3

[11] R. K. Shields , L. J. Enloe , R. E. Evans , K. B. Smith , and S. D. Steckel , “Reliability, Validity, and Responsiveness of Functional Tests in Patients With Total Joint Replacement,” Physical Therapy 75, no. 3 (1995): 169–176 discussion 176–9. 7870749 10.1093/ptj/75.3.169

[12] H. Y. Shi , J. K. Chang , C. Y. Wong , et al., “Responsiveness and Minimal Important Differences After Revision Total Hip Arthroplasty,” BMC Musculoskeletal Disorders 11 (2010): 261. 21070675 10.1186/1471-2474-11-261 PMC2992480

[13] H. L. Hoeksma , C. H. Van Den Ende , H. K. Ronday , A. Heering , and F. C. Breedveld , “Comparison of the Responsiveness of the Harris Hip Score With Generic Measures for Hip Function in Osteoarthritis of the Hip,” Annals of the Rheumatic Diseases 62, no. 10 (2003): 935–938. 12972470 10.1136/ard.62.10.935 PMC1754316

[14] R. Prill , J. Karlsson , O. R. Ayeni , and R. Becker , “Author Guidelines for Conducting Systematic Reviews and Meta‐Analyses,” Knee Surgery, Sports Traumatology, Arthroscopy 29, no. 9 (2021): 2739–2744, 10.1007/s00167-021-06631-7. 34181055

[15] M. J. Page , J. E. McKenzie , P. M. Bossuyt , et al., “The PRISMA 2020 Statement: An Updated Guideline for Reporting Systematic Reviews,” BMJ(Clinical reasearch ed.) 372 (2021): n71, 10.1136/bmj.n71. PMC8005924 33782057

[16] J. A. C. Sterne , J. Savović , M. J. Page , et al., “RoB 2: A Revised Tool for Assessing Risk of Bias in Randomised Trials,” BMJ(Clinical reasearch ed.) 366 (2019): l4898, 10.1136/bmj.l4898. 31462531

[17] G. H. Guyatt , A. D. Oxman , G. E. Vist , et al., “GRADE: An Emerging Consensus on Rating Quality of Evidence and Strength of Recommendations,” BMJ(Clinical reasearch ed.) 336, no. 7650 (2008): 924–926, 10.1136/bmj.39489.470347.AD. PMC2335261 18436948

[18] J. P. Higgins and S. G. Thompson , “Quantifying Heterogeneity in a Meta‐Analysis,” Statistics in Medicine 21, no. 11 (2002): 1539–1558, 10.1002/sim.1186. 12111919

[19] A. Auffarth , H. Resch , S. Lederer , et al., “Does the Choice of Approach for Hip Hemiarthroplasty in Geriatric Patients Significantly Influence Early Postoperative Outcomes? A Randomized‐Controlled Trial Comparing the Modified Smith‐Petersen and Hardinge Approaches,” Journal of Trauma 70, no. 5 (2011): 1257–1262, 10.1097/TA.0b013e3181eded53. 21206288

[20] W. P. Barrett , S. E. Turner , and J. P. Leopold , “Prospective Randomized Study of Direct Anterior vs Postero‐Lateral Approach for Total Hip Arthroplasty,” Journal of Arthroplasty 28, no. 9 (2013): 1634–1638, 10.1016/j.arth.2013.01.034. 23523485

[21] G. Bon , E. B. Kacem , P. M. Lepretre , et al., “Does the Direct Anterior Approach Allow Earlier Recovery of Walking Following Total Hip Arthroplasty? A Randomized Prospective Trial Using Accelerometry,” Orthopaedics & Traumatology, Surgery & Research 105, no. 3 (2019): 445–452, 10.1016/j.otsr.2019.02.008. 30853454

[22] G. H. Dai , Y. Yin , Y. Y. Ji , and S. Q. Yi , “Effect of Artificial Femoral Head Replacement on Senile Osteoporotic Femoral Neck Fracture,” J Trauma Surg 21, no. 10 (2019): 761–765, 10.3969/j.issn.1009-4237.2019.10.011.

[23] C. D'Arrigo , A. Speranza , E. Monaco , A. Carcangiu , and A. Ferretti , “Learning Curve in Tissue Sparing Total Hip Replacement: Comparison Between Different Approaches,” Journal of Orthopaedics and Traumatology 10, no. 1 (2009): 47–54, 10.1007/s10195-008-0043-1. 19384637 PMC2657353

[24] B. De Anta‐Díaz , J. Serralta‐Gomis , A. Lizaur‐Utrilla , E. Benavidez , and F. A. López‐Prats , “No Differences Between Direct Anterior and Lateral Approach for Primary Total Hip Arthroplasty Related to Muscle Damage or Functional Outcome,” International Orthopaedics 40, no. 10 (2016): 2025–2030, 10.1007/s00264-015-3108-9. 26753844

[25] T. Dienstknecht , C. Lüring , M. Tingart , J. Grifka , and E. Sendtner , “A Minimally Invasive Approach for Total Hip Arthroplasty Does Not Diminish Early Post‐Operative Outcome in Obese Patients: A Prospective, Randomised Trial,” International Orthopaedics 37, no. 6 (2013): 1013–1018, 10.1007/s00264-013-1833-5. 23446330 PMC3664165

[26] X. Ding , X. Li , and J. Luo , “Minimally Invasive SuperPath Approach and Traditional Approach in Elderly Patients With Femoral Neck Fractures. Study on the Effect of Efficacy and Expression of Inflammatory Factors After Surgery,” Jiangxi Medical Journal 58, no. 5 (2023): 563–566, 10.3969/j.issn.1006-2238.2023.05.013.

[27] P. Gao and X. Shi , “The Effect of Total Hip Replacement With Minimally Invasive SuperPATH Approach in the Treatment of Femoral Neck Fractures in the Elderly,” Henan Med Res 29, no. 20 (2020): 3715–3717, 10.3969/j.issn.1004-437X.2020.20.024 (In Chinese).

[28] J. Z. Hou , H. Bao , and Y. Cheng , “Early Effect Observation of Total Hip Arthroplasty by Using SuperPATH Technique,” Journal of Clinical Orthopaedics 20 (2017): 50–53, 10.3969/j.issn.1008-0287.2017.01.023 (In Chinese).

[29] K. Huang , K. Xie , Y. Shi , et al., “Analysis of Early Clinical Efficacy of SuperPATH Approach and Lateral Approach for Initial Total Hip Arthroplasty,” Youjiang Med J 49, no. 9 (2021): 646–651, 10.3969/j.issn.1003-1383.2021.09.002 (In Chinese).

[30] J. Jia , B. Yu , L. Wu , Z. Zhi , and L. Pan , “Hip Hemiarthroplasty for Senile Femoral Neck Fractures: Minimally Invasive SuperPath Approach Versus Traditional Posterior Approach,” Chininese. Journal Geriatric Orthopaedics and Rehabilitation 3, no. 4 (2017): 223–231, 10.3877/cma.j.issn.2096-0263.2017.04.006.

[31] L. Li , “SuperPATH Minimally Invasive Total Hip Replacement Surgery Treatment: Analysis of Clinical Efficacy of Aseptic Necrosis of Femoral Head,” Chin J Mod Drug Appl 14, no. 12 (2020): 84–86, 10.14164/j.cnki.cn11-5581/r.2020.12.039 (In Chinese).

[32] M. Li , “VAS Score of Patients With Femoral Neck Fractures Treated With SuperPATH Approach Hip Arthroplasty and Impact on Hip Joint Recovery,” Practical Clinical Integration of Traditional Chinese and Western Medicine 21, no. 13 (2021): 28–29, 10.13638/j.issn.1671-4040.2021.13.013.

[33] Q. Q. Li , Z. Zhang , B. Liu , Y. Z. Wang , and X. Zhai , “Comparison of Two Approaches of Total Hip Arthroplasty for Femoral Head Necrosis,” Orthopedic Journal of China 31, no. 21 (2023): 1956–1960, 10.3977/j.issn.1005-8478.2023.21.06.

[34] Z. Ling , P. Zhou , and Y. Fu , “Analysis of the Effect of Total Hip Replacement via SuperPATH Approach on the Prognosis of Elderly Patients With Femoral Neck Fracture,” Chin. J. Front. Med. Sci 12, no. 5 (2020): 66–70, 10.12037/YXQY.2020.05-10 (In Chinese).

[35] W. Liu , X. Liu , H. Gao , G. Wang , and J. Li , “Comparison of the Curative Effect, Pain Degree, and Hip Joint Function Between SuperPATH Hip Replacement and Total Hip Replacement,” Mod Chin Doc 60, no. 6 (2022): 78–84 (In Chinese).

[36] Y. Liu , P. Hu , J. Zhu , H. She , and Y. Zhang , “Efficacy of Minimally Invasive Total Hip Arthroplasty in the Treatment of Elderly Femoral Neck Fractures,” Prac. J. Med. Pharm 38, no. 3 (2021): 226–231, 10.14172/j.issn1671-4008.2021.03.010 (In Chinese).

[37] R. Martin , P. E. Clayson , S. Troussel , B. P. Fraser , and P. L. Docquier , “Anterolateral Minimally Invasive Total Hip Arthroplasty: A Prospective Randomized Controlled Study With a Follow‐Up of 1 Year,” Journal of Arthroplasty 26, no. 8 (2011): 1362–1372, 10.1016/j.arth.2010.11.016. 21435823

[38] W. Meng , Z. Huang , H. Wang , et al., “Supercapsular Percutaneously‐Assisted Total Hip (SuperPath) Versus Posterolateral Total Hip Arthroplasty in Bilateral Osteonecrosis of the Femoral Head: A Pilot Clinical Trial,” BMC Musculoskeletal Disorders 21, no. 1 (2019): 2, 10.1186/s12891-019-3023-0. 31892355 PMC6937651

[39] K. Moerenhout , P. Derome , G. Y. Laflamme , S. Leduc , H. S. Gaspard , and B. Benoit , “Direct Anterior Versus Posterior Approach for Total Hip Arthroplasty: A Multicentre, Prospective, Randomized Clinical Trial,” Canadian Journal of Surgery 63, no. 5 (2020): E412–E417, 10.1503/cjs.012019. PMC7608717 33009898

[40] M. Müller , S. Tohtz , I. Springer , M. Dewey , and C. Perka , “Randomized Controlled Trial of Abductor Muscle Damage in Relation to the Surgical Approach for Primary Total Hip Replacement: Minimally Invasive Anterolateral Versus Modified Direct Lateral Approach,” Archives of Orthopaedic and Trauma Surgery 131, no. 2 (2011): 179–189, 10.1007/s00402-010-1117-0. 20490520

[41] C. Ouyang , H. Wang , W. Meng , et al., “Randomized Controlled Trial of Comparison Between the SuperPATH and Posterolateral Approaches in Total Hip Arthroplasty,” Zhongguo Xiu Fu Chong Jian Wai Ke Za Zhi 32, no. 12 (2018): 1500–1506, 10.7507/1002-1892.201807011 (In Chinese). 30569673 PMC8414232

[42] Y. Pan , J. Zhang , X. Yan , X. Chang , J. Li , and B. Tang , “Comparison of SuperPATH and Posterolateral Total Hip Replacement,” Orthop. J. China 28 (2020): 1176–1180 (In Chinese).

[43] J. C. Reichert , E. von Rottkay , F. Roth , et al., “A Prospective Randomized Comparison of the Minimally Invasive Direct Anterior and the Transgluteal Approach for Primary Total Hip Arthroplasty,” BMC Musculoskeletal Disorders 19, no. 1 (2018): 241, 10.1186/s12891-018-2133-4. 30025519 PMC6053824

[44] D. Ren , G. Yang , H. Zhao , J. Zha , S. Lu , and Y. Xu , “Effect of SuperPath Minimally Invasive Incision Total Hip Arthroplasty on Femoral Head Necrosis and the Quality of Life,” Journal of Hebei Medical University 37, no. 12 (2016): 1416–1419, 10.3969/j.issn.1007-3205.2016.12.013 (In Chinese).

[45] C. Restrepo , J. Parvizi , A. E. Pour , and W. J. Hozack , “Prospective Randomized Study of Two Surgical Approaches for Total Hip Arthroplasty,” Journal of Arthroplasty 25, no. 5 (2010): 671–679, 10.1016/j.arth.2010.02.002. 20378307

[46] K. Rykov , I. H. F. Reininga , M. S. Sietsma , B. A. S. Knobben , and B. L. E. F. Ten Have , “Posterolateral vs Direct Anterior Approach in Total Hip Arthroplasty (POLADA Trial): A Randomized Controlled Trial to Assess Differences in Serum Markers,” Journal of Arthroplasty 32, no. 12 (2017): 3652–3658.e1, 10.1016/j.arth.2017.07.008. 28780222

[47] M. Schwarze , S. Budde , G. von Lewinski , et al., “No Effect of Conventional vs. Minimally Invasive Surgical Approach on Clinical Outcome and Migration of a Short Stem Total Hip Prosthesis at 2‐Year Follow‐Up: A Randomized Controlled Study,” Clinical Biomechanics (Bristol, Avon) 51 (2018): 105–112, 10.1016/j.clinbiomech.2017.12.004. 29287171

[48] M. J. Taunton , J. B. Mason , S. M. Odum , and B. D. Springer , “Direct Anterior Total Hip Arthroplasty Yields More Rapid Voluntary Cessation of all Walking Aids: A Prospective, Randomized Clinical Trial,” Journal of Arthroplasty 29 (2014): 169–172, 10.1016/j.arth.2014.03.051. 25007723

[49] M. J. Taunton , R. T. Trousdale , R. J. Sierra , K. Kaufman , and M. W. Pagnano , “John Charnley Award: Randomized Clinical Trial of Direct Anterior and Miniposterior Approach THA: Which Provides Better Functional Recovery?,” Clinical Orthopaedics and Related Research 476, no. 2 (2018): 216–229, 10.1007/s11999.0000000000000112. 29529650 PMC6259722

[50] J. R. Varela Egocheaga , M. Á. Suárez‐Suárez , M. Fernández‐Villán , V. González‐Sastre , J. Varela‐Gómez , and A. Murcia‐Mazón , “Abordaje Posterior mínimamente Invasivo en Artroplastia Total de Cadera. Estudio Prospectivo y Aleatorizado. Un año de Seguimiento [Minimally Invasive Posterior Approach in Total Hip Arthroplasty. Prospective Randomised Trial],” Anales del Sistema Sanitario de Navarra 33, no. 2 (2010): 133–143. (Spanish), 10.4321/s1137-66272010000300002. 20927140

[51] X. Wang and J. Tian , “Minimally Invasive Femoral Head Replacement for Femoral Neck Fractures in the Elderly. Study on the Influence of Postoperative Hip Joint Mobility,” Guizou Medical Journal 45, no. 5 (2021): 780–782.

[52] Z. Wang and W. Ge , “SuperPATH Approach Total Hip Replacement for Elderly Patients With Femoral Neck Fracture: Impact of Hip Function,” Clinical Medicine 41, no. 1 (2021): 27–29, 10.19528/j-issn.1003-3548.2021.01.010 (In Chinese).

[53] J. Xie , H. Zhang , L. Wang , X. Yao , Z. Pan , and Q. Jiang , “Comparison of Supercapsular Percutaneously Assisted Approach Total Hip Versus Conventional Posterior Approach for Total Hip Arthroplasty: A Prospective, Randomized Controlled Trial,” Journal of Orthopaedic Surgery and Research 12, no. 1 (2017): 138, 10.1186/s13018-017-0636-6. 28946892 PMC5613398

[54] G. Xu , L. Hu , and S. Yang , “SuperPATH Minimally Invasive Approach to Artificial Femoral Head Replacement. A Short‐Term Follow‐Up Study on the Treatment of Femoral Neck Fractures in the Elderly,” Hainan Medical Journal (Hai Nan Yi Xue) 29, no. 17 (2018): 2400–2404, 10.3969/j.issn.1003-6350.2018.17.010.

[55] T. Yan , S. Tian , Y. Wang , et al., “Comparison of Early Effectiveness Between SuperPATH Approach and Hardinge Approach in Total Hip Arthroplasty,” Zhongguo Xiu Fu Chong Jian Wai Ke Za Zhi 31, no. 1 (2017): 17–24, 10.7507/1002-1892.201609110 (In Chinese). 29798623 PMC9548045

[56] C. Yang , Q. Zhu , Y. Han , et al., “Minimally‐Invasive Total Hip Arthroplasty Will Improve Early Postoperative Outcomes: A Prospective, Randomized, Controlled Trial,” Irish Journal of Medical Science 179, no. 2 (2010): 285–290, 10.1007/s11845-009-0437-y. 19847593

[57] H. Yuan , J. Zhu , Z. Sun , and Z. Zhang , “Comparison of Effectiveness Between SuperPATH Approach and Posterolateral Approach in Total Hip Arthroplasty,” Zhongguo Xiu Fu Chong Jian Wai Ke Za Zhi 32, no. 1 (2018): 14–19, 10.7507/1002-1892.201707121 (In Chinese). 29806358 PMC8414201

[58] Z. L. Zhang , J. H. Lin , and B. Xia , “Clinical Research on Joint Function and Life Quality Through SuperPath Approach in Total Hip Arthroplasty,” China J Integrated Trad Chin Western Med 25, no. 5 (2019): 709–714, 10.3969/j.issn.1007-6948.2019.05.012.

[59] H. Y. Zhao , P. D. Kang , Y. Y. Xia , X. J. Shi , Y. Nie , and F. X. Pei , “Comparison of Early Functional Recovery After Total Hip Arthroplasty Using a Direct Anterior or Posterolateral Approach: A Randomized Controlled Trial,” Journal of Arthroplasty 32, no. 11 (2017): 3421–3428, 10.1016/j.arth.2017.05.056. 28662957

[60] M. Chang , X. Liu , Y. Feng , and Z. Liu , “Treatment of Bipolar Femoral Head Replacement With Modified SuperPATH Approach: Early and Mid‐Term Curative Effect Analysis of Femoral Neck Fracture in the Elderly,” Chin. J. Clin 50, no. 4 (2022): 465–468, 10.3969/j.issn.2095-8552.2022.04.027.

[61] Y. Ding , “Minimally Invasive SuperPath Approach Artificial Femoral Head Replacement in the Treatment of Femoral Neck Fractures in the Elderly,” Shenzhen J. Int. Med 28, no. 16 (2018): 129–131, 10.16458/j.cnki.1007-0893.2018.16.064.

[62] Y. Li , Z. K. He , X. M. Guo , X. Sun , and Y. Yang , “Effects of Artificial Femoral Head Replacement Between SuperPath Approach and Small Incision Posterolateral Approach on Elderly Patients With Femoral Neck Fractures,” J. Rare Uncommon Dis 26, no. 5 (2019): 66–75, 10.3969/j.issn.1009-3257.2019.05.024.

[63] M. Tian , Y. Gao , W. Wu , and J. Shu , “SuperPATH Approach for Hip Arthroplasty in the Treatment of Femoral Neck Fractures: Effectiveness and Effect on Complication Rate,” Chin. J. Clin 48, no. 1 (2020): 82–84, 10.3969/j.issn.2095-8552.2020.01.025.

[64] G. H. Wu , Y. Di , Y. H. Ma , J. L. Zhao , and Y. H. Liang , “Short‐Term Efficacy of SuperPATH Approach for Hip Arthroplasty in the Elderly With Femoral Neck Fracture,” Chin. J. Mult. Organ Dis. Elder 17, no. 7 (2018): 529–532, 10.11915/j.issn.1671-5403.2018.07.119.

[65] H. Aiba , N. Watanabe , Y. Nishimori , et al., “Randomized Study of Direct Anterior Approach Versus Posterior Approach for Bipolar Hemiarthroplasty of the Hip,” J Ortho Rheum 1, no. 2 (2015): 10.

[66] B. H. Brismar , O. Hallert , A. Tedhamre , and J. U. Lindgren , “Early Gain in Pain Reduction and Hip Function, but More Complications Following the Direct Anterior Minimally Invasive Approach for Total Hip Arthroplasty: A Randomized Trial of 100 Patients With 5 Years of Follow Up,” Acta Orthopaedica 89, no. 5 (2018): 484–489, 10.1080/17453674.2018.1504505. 30350758 PMC6202757

[67] O. L. Brun , H. N. Sund , L. Nordsletten , S. M. Röhrl , and K. E. Mjaaland , “Component Placement in Direct Lateral vs Minimally Invasive Anterior Approach in Total Hip Arthroplasty: Radiographic Outcomes From a Prospective Randomized Controlled Trial,” Journal of Arthroplasty 34, no. 8 (2019): 1718–1722, 10.1016/j.arth.2019.04.003. 31053468

[68] G. Bűcs , Á. Dandé , B. Patczai , et al., “Bipolar Hemiarthroplasty for the Treatment of Femoral Neck Fractures With Minimally Invasive Anterior Approach in Elderly,” Injury 52, no. Suppl 1 (2021): S37–S43, 10.1016/j.injury.2020.02.053. 32115214

[69] T. E. Cheng , J. A. Wallis , N. F. Taylor , et al., “A Prospective Randomized Clinical Trial in Total Hip Arthroplasty‐Comparing Early Results Between the Direct Anterior Approach and the Posterior Approach,” Journal of Arthroplasty 32 (2017): 883–890, 10.1016/j.arth.2016.08.027. 27687805

[70] C. P. Christensen and C. A. Jacobs , “Comparison of Patient Function During the First Six Weeks After Direct Anterior or Posterior Total Hip Arthroplasty (THA): A Randomized Study,” Journal of Arthroplasty 30, no. 9 Suppl (2015): 94–97, 10.1016/j.arth.2014.12.0382017. 26096071

[71] H. J. Cooper , W. M. Santos , A. L. Neuwirth , et al., “Randomized Controlled Trial of Incisional Negative Pressure Following High‐Risk Direct Anterior Total Hip Arthroplasty,” Journal of Arthroplasty 37, no. 8S (2022): S931–S936, 10.1016/j.arth.2022.03.039. 35304299

[72] B. Ding , F. Bao , X. Chen , et al., “Minimally Invasive SuperPath Approach Versus Conventional Approach in Elderly Patients With Femoral Neck Fracture,” J J Traumatic 23, no. 3 (2018): 471–472, 10.3969/j.issn.1009-7147.2018.03.026.

[73] A. M. Fahs , D. M. Koueiter , M. D. Kurdziel , K. A. Huynh , C. R. Perry , and J. J. Verner , “Psoas Compartment Block vs Periarticular Local Anesthetic Infiltration for Pain Management After Anterior Total Hip Arthroplasty: A Prospective, Randomized Study,” J Arthroplasty 33, no. 7 (2018): 2192–2196, 10.1016/j.arth.2018.02.052. 29555492

[74] A. Fraval , P. Effeney , L. Fiddelaers , B. Smith , B. Towell , and P. Tran , “OBTAIN A: Outcome Benefits of Tranexamic Acid in Hip Arthroplasty. A Randomized Double‐Blinded Controlled Trial,” Journal of Arthroplasty 32, no. 5 (2017): 1516–1519, 10.1016/j.arth.2016.11.045. 28089468

[75] A. Fraval , S. Duncan , T. Murray , J. Duggan , O. Tirosh , and P. Tran , “OBTAIN E: Outcome Benefits of Tranexamic Acid in Hip Arthroplasty With Enoxaparin: A Randomised Double‐Blinded Controlled Trial,” Hip International 29, no. 3 (2019): 239–244, 10.1177/1120700018780125. 30039736

[76] Q. Gong , W. Gong , G. Zhou , et al., “SuperPATH Approach Artificial Femoral Head Replacement Combined With Traditional Chinese Medicine in the Treatment of Elderly Femoral Neck,” Pract Int Trad Chin West Med 18, no. 8 (2018): 36–38, 10.13638/j.issn.1671-4040.2018.08.016.

[77] R. Iorio , E. Viglietta , D. Mazza , et al., “Do Serum Markers Correlate With Invasiveness of the Procedure in THA? A Prospective Randomized Study Comparing Direct Anterior and Lateral Approaches,” Orthopaedics & Traumatology, Surgery & Research 107, no. 8 (2021): 102937. 10.1016/j.otsr.2021.102937 33895386

[78] R. Krassnig , W. Prager , R. Wildburger , and G. M. Hohenberger , “Direct Anterior Versus Antero‐Lateral Approach in Hip Joint Hemiarthroplasty,” Archives of Orthopaedic and Trauma Surgery 143, no. 7 (2023): 4141–4148, 10.1007/s00402-022-04685-x. 36394659

[79] S. Landgraeber , H. Quitmann , S. Güth , et al., “A Prospective Randomized Peri‐ and Post‐Operative Comparison of the Minimally Invasive Anterolateral Approach Versus the Lateral Approach,” Orthop Rev (Pavia) 5, no. 3 (2013): e19, 10.4081/or.2013.e19. 24191179 PMC3808794

[80] Y. Luo , Y. Releken , D. Yang , Y. Yue , Z. Liu , and P. Kang , “Effects of Carbazochrome Sodium Sulfonate Combined With Tranexamic Acid on Hemostasis and Inflammation During Perioperative Period of Total Hip Arthroplasty: A Randomized Controlled Trial,” Orthopaedics & Traumatology, Surgery & Research 108, no. 1 (2022): 103092, 10.1016/j.otsr.2021.103092. 34601160

[81] K. E. Mjaaland , K. Kivle , S. Svenningsen , A. H. Pripp , and L. Nordsletten , “Comparison of Markers for Muscle Damage, Inflammation, and Pain Using Minimally Invasive Direct Anterior Versus Direct Lateral Approach in Total Hip Arthroplasty: A Prospective, Randomized, Controlled Trial,” Journal of Orthopaedic Research 33, no. 9 (2015): 1305–1310, 10.1002/jor.22911. 25877694

[82] K. E. Mjaaland , K. Kivle , S. Svenningsen , and L. Nordsletten , “Do Postoperative Results Differ in a Randomized Trial Between a Direct Anterior and a Direct Lateral Approach in THA?,” Clinical Orthopaedics and Related Research 477, no. 1 (2019): 145–155, 10.1097/CORR.0000000000000439. 30179928 PMC6345297

[83] K. Moerenhout , B. Benoit , H. S. Gaspard , D. M. Rouleau , and G. Y. Laflamme , “Greater Trochanteric Pain After Primary Total Hip Replacement, Comparing the Anterior and Posterior Approach: A Secondary Analysis of a Randomized Trial,” Orthopaedics & Traumatology, Surgery & Research 107, no. 8 (2021): 102709, 10.1016/j.otsr.2020.08.011. 33132093

[84] S. M. J. Mortazavi , M. Razzaghof , E. Ghadimi , S. M. M. Seyedtabaei , M. Vahedian Ardakani , and A. Moharrami , “The Efficacy of Bone Wax in Reduction of Perioperative Blood Loss in Total Hip Arthroplasty via Direct Anterior Approach: A Prospective Randomized Clinical Trial,” Journal of Bone and Joint Surgery. American Volume 104, no. 20 (2022): 1805–1813, 10.2106/JBJS.22.00376. 35984033

[85] M. Nambiar , T. E. Cheng , J. R. Onggo , et al., “No Difference in Functional, Radiographic, and Survivorship Outcomes Between Direct Anterior or Posterior Approach THA: 5‐Year Results of a Randomized Trial,” Clinical Orthopaedics and Related Research 479, no. 12 (2021): 2621–2629, 10.1097/CORR.0000000000001855. 34237041 PMC8726547

[86] D. V. Nistor , S. Caterev , S. D. Bolboacă , D. Cosma , D. O. G. Lucaciu , and A. Todor , “Transitioning to the Direct Anterior Approach in Total Hip Arthroplasty. Is It a True Muscle Sparing Approach When Performed by a Low Volume Hip Replacement Surgeon?,” International Orthopaedics 41, no. 11 (2017): 2245–2252, 10.1007/s00264-017-3480-8. 28439629

[87] J. Parvizi , C. Restrepo , and M. G. Maltenfort , “Total Hip Arthroplasty Performed Through Direct Anterior Approach Provides Superior Early Outcome: Results of a Randomized, Prospective Study,” Orthop Clin North Am 47, no. 3 (2016): 497–504, 10.1016/j.ocl.2016.03.003. 27241374

[88] C. R. Perry, Jr. , A. M. Fahs , M. D. Kurdziel , D. M. Koueiter , R. J. Fayne , and J. J. Verner , “Intraoperative Psoas Compartment Block vs Preoperative Fascia Iliaca Block for Pain Control After Direct Anterior Total Hip Arthroplasty: A Randomized Controlled Trial,” Journal of Arthroplasty 33, no. 6 (2018): 1770–1774, 10.1016/j.arth.2018.01.010. 29615378

[89] I. H. Reininga , M. Stevens , R. Wagenmakers , et al., “Comparison of Gait in Patients Following a Computer‐Navigated Minimally Invasive Anterior Approach and a Conventional Posterolateral Approach for Total Hip Arthroplasty: A Randomized Controlled Trial,” Journal of Orthopaedic Research 31, no. 2 (2013): 288–294, 10.1002/jor.22210. 22886805

[90] F. Renken , S. Renken , A. Paech , M. Wenzl , A. Unger , and A. P. Schulz , “Early Functional Results After Hemiarthroplasty for Femoral Neck Fracture: A Randomized Comparison Between a Minimal Invasive and a Conventional Approach,” BMC Musculoskeletal Disorders 8, no. 13 (2012): 141, 10.1186/1471-2474-13-141. PMC3488324 22873207

[91] M. Sancheti and M. Ghagre , “Bipolar Hemiarthroplasty of Hip Joint: Prospective Randomised Comparative Study of Direct Anterior Approach Versus Posterior Approach,” International Journal of Research Orthopaedics 7, no. 2 (2021): 381–385, 10.18203/issn.2455-4510.

[92] W. Sang , S. Xue , Y. Xu , Y. Liu , L. Zhu , and J. Ma , “Bikini Incision Increases the Incidence of Lateral Femoral Cutaneous Nerve Injury in Direct Anterior Approach Hip Arthroplasty: A Prospective Ultrasonic, Electrophysiological, and Clinical Study,” Journal of Arthroplasty 36, no. 10 (2021): 3463–3470, 10.1016/j.arth.2021.05.012. 34074541

[93] F. Saxer , P. Studer , M. Jakob , et al., “Minimally Invasive Anterior Muscle‐Sparing Versus a Transgluteal Approach for Hemiarthroplasty in Femoral Neck Fractures‐a Prospective Randomised Controlled Trial Including 190 Elderly Patients,” BMC Geriatrics 18, no. 1 (2018): 222, 10.1186/s12877-018-0898-9. 30241509 PMC6151034

[94] A. M. Schwartz , R. K. Goel , A. P. Sweeney , and T. L. Bradbury, Jr. , “Capsular Management in Direct Anterior Total hip Arthroplasty: A Randomized, Single‐Blind, Controlled Trial,” J Arthroplasty 36, no. 8 (2021): 2836–2842, 10.1016/j.arth.2021.03.048. 33865648

[95] J. C. Suarez , E. M. Slotkin , C. R. Szubski , W. K. Barsoum , and P. D. Patel , “Prospective, Randomized Trial to Evaluate Efficacy of a Bipolar Sealer in Direct Anterior Approach Total Hip Arthroplasty,” Journal of Arthroplasty 30, no. 11 (2015): 1953–1958, 10.1016/j.arth.2015.05.023. 26093486

[96] W. Sun , “The Comparison Effect of Total Hip Arthroplasty via Two Kind of Approaches for Femoral Neck Fracture,” Journal of Clinical Orthopaedics 27, no. 1 (2023): 35–39, 10.3969/j.issn.1008.0287.2024.01.011.

[97] R. Takada , T. Jinno , K. Miyatake , et al., “Direct Anterior Versus Anterolateral Approach in One‐Stage Supine Total Hip Arthroplasty. Focused on Nerve Injury: A Prospective, Randomized, Controlled Trial,” Journal of Orthopaedic Science 23, no. 5 (2018): 783–787, 10.1016/j.jos.2018.05.005. 29935972

[98] M. Thaler , R. Lechner , D. Putzer , et al., “Two‐Year Gait Analysis Controls of the Minimally Invasive Total Hip Arthroplasty by the Direct Anterior Approach,” Clinical Biomechanics (Bristol, Avon) 58 (2018): 34–38, 10.1016/j.clinbiomech.2018.06.018. 30015203

[99] S. Verzellotti , C. Candrian , M. Molina , G. Filardo , R. Alberio , and F. A. Grassi , “Direct Anterior Versus Posterolateral Approach for Bipolar Hip Hemiarthroplasty in Femoral Neck Fractures: A Prospective Randomised Study,” Hip International 30, no. 6 (2020): 810–817, 10.1177/1120700019872117. 31450987

[100] G. F. Vles , K. Corten , R. Driesen , C. van Elst , and S. G. Ghijselings , “Hidden Blood Loss in Direct Anterior Total Hip Arthroplasty: A Prospective, Double Blind, Randomized Controlled Trial on Topical Versus Intravenous Tranexamic Acid,” Musculoskeletal Surgery 105, no. 3 (2021): 267–273, 10.1007/s12306-020-00652-0. 32152813

[101] Q. Wang , Y. Yue , Z. Yang , L. Chen , Q. Li , and P. Kang , “Comparison of Postoperative Outcomes Between Traditional Longitudinal Incision and Bikini Incision in Total Hip Arthroplasty via Direct Anterior Approach: A Randomized Controlled Trial,” Journal of Arthroplasty 36, no. 1 (2021): 222–230, 10.1016/j.arth.2020.07.047. 32800438

[102] N. Watanabe , H. Aiba , and G. Sagara , “Prospective Randomised Study of Direct Anterior Approach Versus Posterior Approach for Bipolar Hemiarthroplasty of the Hip,” Orthopeadic Proceedings 98 (2016): 123.

[103] L. Z. Xia , S. H. Li , Z. S. Yuan , et al., “Common Bipolar Femoral Head by SuperPATH Approach for Senile Femoral Neck Fractures,” Chin J Tiss Eng Res 22, no. 19 (2018): 2953–2960, 10.3969/j.issn.2095-4344.0282.

[104] C. Xiao , Z. Gao , S. Zhang , et al., “Comparative Prospective Randomized Study of Minimally Invasive Transpiriformis Approach Versus Conventional Posterolateral Approach in Total Hip Arthroplasty as Measured by Biology Markers,” International Orthopaedics 45, no. 7 (2021): 1707–1717, 10.1007/s00264-021-05083-5. 34043029 PMC8266695

[105] Z. Yang , S. Feng , K. J. Guo , and G. C. Zha , “Patient‐Reported Results of Simultaneous Direct Anterior Approach and Posterolateral Approach Total Hip Arthroplasties Performed in the Same Patients,” Journal of Orthopaedics and Traumatology 22, no. 1 (2021): 46, 10.1186/s10195-021-00611-w. 34773489 PMC8590638

[106] H. Y. Zhao , M. Xiang , Y. Xia , X. Shi , F. X. Pei , and P. Kang , “Efficacy of Oral Tranexamic Acid on Blood Loss in Primary Total Hip Arthroplasty Using a Direct Anterior Approach: A Prospective Randomized Controlled Trial,” International Orthopaedics 42, no. 11 (2018): 2535–2542, 10.1007/s00264-018-3846-6. 29492612

[107] L. Zhao , Q. Li , and B. Xu , “Treatment of Hemiarthroplasty With SuperPATH Minimally Invasive Approach: Clinical Curative Effect Analysis of Femoral Neck Fracture in Elderly Patients,” Contemp Med 25, no. 34 (2019): 144–146, 10.3969/j.issn.1009-4393.2019.34.060.

[108] S. Zhao , “Minimally Invasive SuperPATH Approach for Hip Replacement in Elderly Patients. Analysis of Clinical Efficacy in Patients With Bone Neck Fractures,” Mod Diagn Treat 32, no. 22 (2021): 3593–3594.

[109] J. Zhang , “Minimally Invasive SuperPATH Approach Total Hip Replacement for the Treatment of Femoral Neck Fractures in the Elderly,” Discussion on the Short‐Term Efficacy and Safety 36, no. 1 (2023): 58–59, 10.3969/j.issn.2095-9559.2023.01.30.

[110] B. U. Nwachukwu , B. Chang , B. Z. Rotter , B. T. Kelly , A. S. Ranawat , and D. H. Nawabi , “Minimal Clinically Important Difference and Substantial Clinical Benefit After Revision Hip Arthroscopy,” Arthroscopy 34, no. 6 (2018): 1862–1868, 10.1016/j.arthro.2018.01.050. 29653791

