# Supplementary figures and images for: Postoperative Harris Hip Score Versus Harris Hip Score Difference in Hip Replacement: What to Report?
Source: Orthop Surg. 2024 Oct 21;17(1):3–21. doi: 10.1111/os.14272 (PMC11735366; doi:10.1111/os.14272)

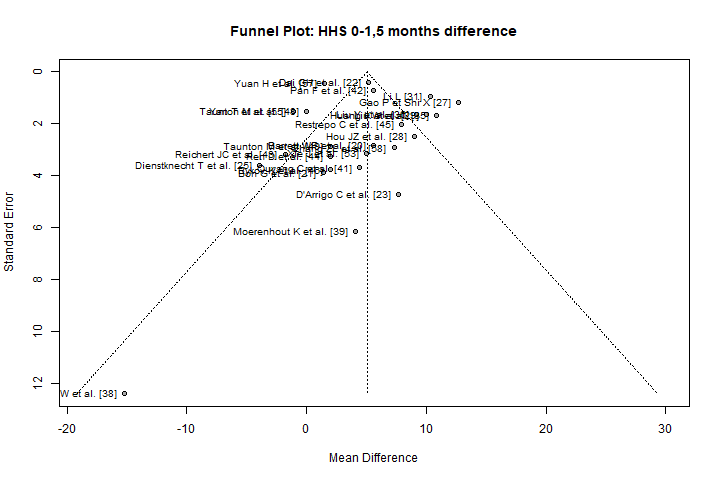

Supplement: Supplementary file 1 — Data S1. Supporting Information. [file OS-17-3-s001.zip › 3_Funnelplot_HHS 0-1,5 months difference.png]

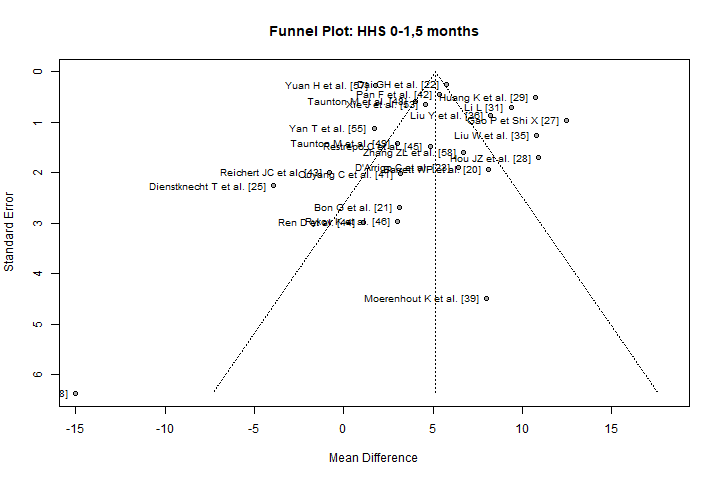

Supplement: Supplementary file 1 — Data S1. Supporting Information. [file OS-17-3-s001.zip › 3_Funnelplot_HHS 0-1,5 months.png]

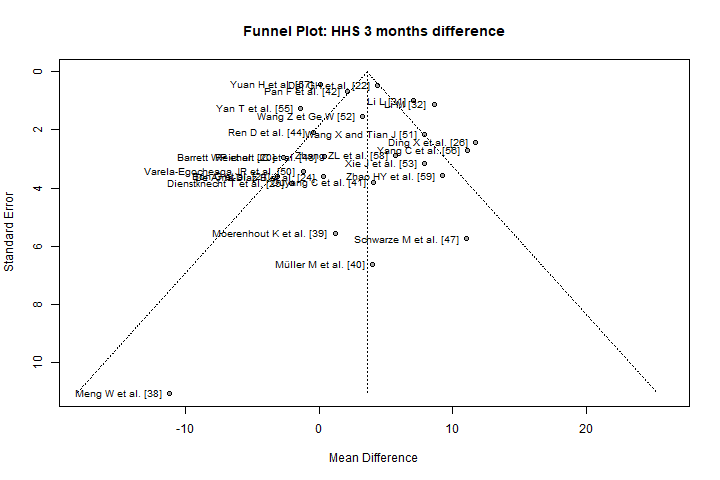

Supplement: Supplementary file 1 — Data S1. Supporting Information. [file OS-17-3-s001.zip › 3_Funnelplot_HHS 3 months difference.png]

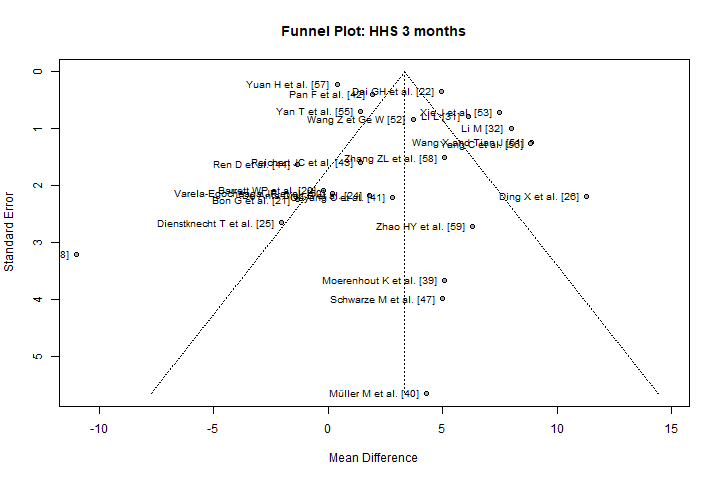

Supplement: Supplementary file 1 — Data S1. Supporting Information. [file OS-17-3-s001.zip › 3_Funnelplot_HHS 3 months.png]

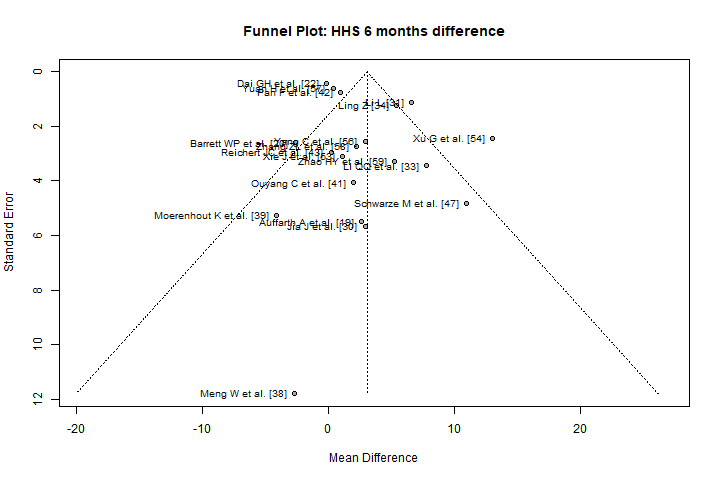

Supplement: Supplementary file 1 — Data S1. Supporting Information. [file OS-17-3-s001.zip › 3_Funnelplot_HHS 6 months difference.png]

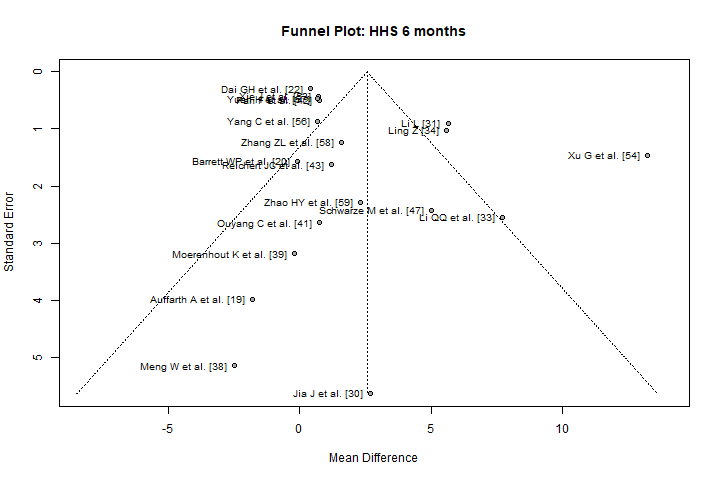

Supplement: Supplementary file 1 — Data S1. Supporting Information. [file OS-17-3-s001.zip › 3_Funnelplot_HHS 6 months.png]

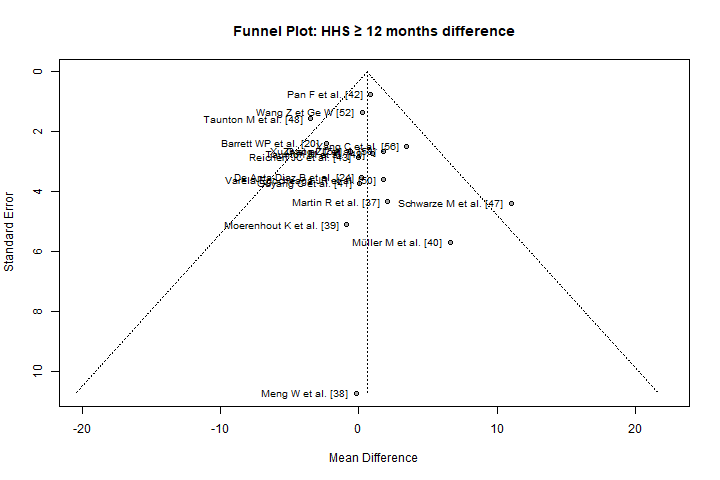

Supplement: Supplementary file 1 — Data S1. Supporting Information. [file OS-17-3-s001.zip › 3_Funnelplot_HHS ≥ 12 months difference.png]

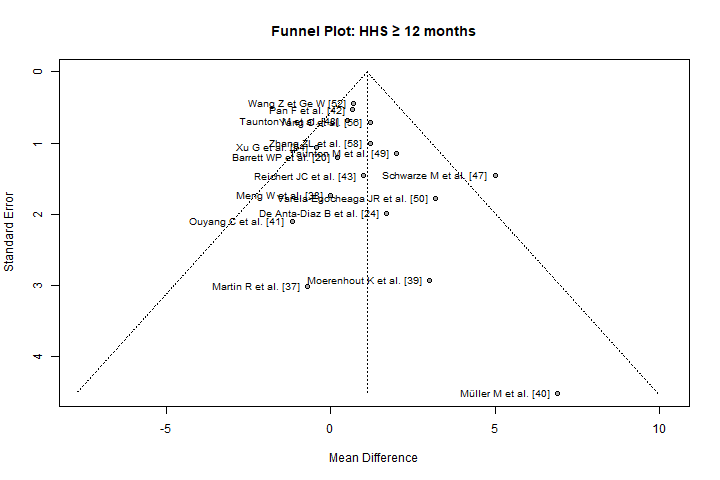

Supplement: Supplementary file 1 — Data S1. Supporting Information. [file OS-17-3-s001.zip › 3_Funnelplot_HHS ≥ 12 months.png]
